# Supplementary material for: Effects of High-Intensity Interval Training Using the 3/7 Resistance Training Method on Metabolic Stress in People with Heart Failure and Coronary Artery Disease: A Randomized Cross-Over Study
Source: J Clin Med. 2023 Dec 17;12(24):7743. doi: 10.3390/jcm12247743 (PMC10743906; doi:10.3390/jcm12247743)
Supplement: Supplementary file 1 [file jcm-12-07743-s001.zip › jcm-2756626-supplementary.pdf]

Supplementary Material

Table S1. Iron metabolism; numerical results of metabolites evolution.

	HFrEF n=9	CAD n=6	P-value
**Time since diagnosis (days) ***	558 (168-2666)	101 (82-136)	<0.01
**Creatine**	1.16 (0.3)	0.95 (0.08)	0.09
**Hemoglobin, g·L−1**	14.2 (1)	14.2 (2)	0.782
**Serum iron, µg·dL−1**	77 (33)	86 (41)	0.723
**Transferrin, g·L−1**	232 (25)	262 (53)	0.391
**Transferrin sat, %**	27 (13)	29 (8)	0.894
**Total iron binding capacity (µg·dL−1**	297 (25)	282 (157)	0.641

Values are mean ± SD

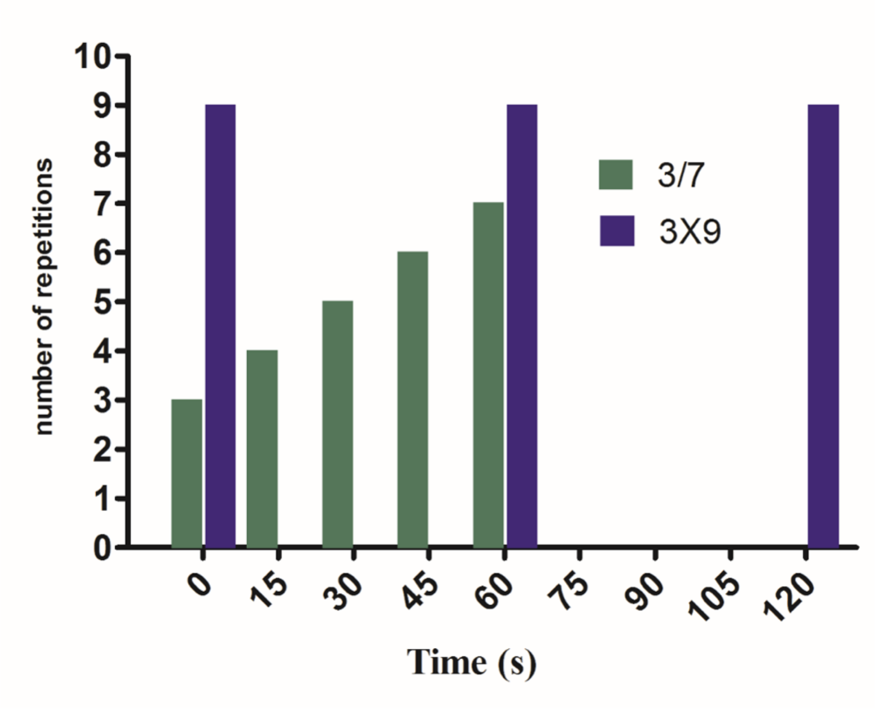

Figure S1. Organization of 3/7 and 3X9 method of resistance training.
